# Perioperative goal-directed hemodynamic therapy based on radial arterial pulse pressure variation and continuous cardiac index trending reduces postoperative complications after major abdominal surgery: a multi-center, prospective, randomized study

**DOI:** 10.1186/cc12885

**Published:** 2013-09-08

**Authors:** Cornelie Salzwedel, Jaume Puig, Arne Carstens, Berthold Bein, Zsolt Molnar, Krisztian Kiss, Ayyaz Hussain, Javier Belda, Mikhail Y Kirov, Samir G Sakka, Daniel A Reuter

**Affiliations:** 1 Department of Anesthesiology, Center of Anesthesiology and Intensive Care Medicine, University Hospital Hamburg-Eppendorf, Martinistraße 52, 20246 Hamburg, Germany; 2 Department of Anesthesia and Critical Care, Hospital Clínico Universitario, University of Valencia, Avinguda de Blasco Ibáñez, 17, 46010 Valencia, Spain; 3 Department of Anesthesiology and Intensive Care Medicine, University Hospital Schleswig-Holstein, Campus Kiel, Arnold-Heller-Straße 3, 24105 Kiel, Germany; 4 Department of Anesthesiology and Intensive Therapy, University of Szeged, 6. Semmelweis Street, 6725 Szeged, Hungary; 5 Department of Anesthesiology and Intensive Care Medicine, Northern State Medical University, 51 Troitsky Prospect, Arkhangelsk 163061 Russian Federation; 6 Department of Anesthesiology and Operative Intensive Care Medicine, Medical Centre Cologne -Merheim, University Witten/Herdecke, Ostmerheimer Street 200, 51109 Köln, Germany

## Abstract

**Introduction:**

Several single-center studies and meta-analyses have shown that perioperative goal-directed therapy may significantly improve outcomes in general surgical patients. We hypothesized that using a treatment algorithm based on pulse pressure variation, cardiac index trending by radial artery pulse contour analysis, and mean arterial pressure in a study group (SG), would result in reduced complications, reduced length of hospital stay and quicker return of bowel movement postoperatively in abdominal surgical patients, when compared to a control group (CG).

**Methods:**

160 patients undergoing elective major abdominal surgery were randomized to the SG (79 patients) or to the CG (81 patients). In the SG hemodynamic therapy was guided by pulse pressure variation, cardiac index trending and mean arterial pressure. In the CG hemodynamic therapy was performed at the discretion of the treating anesthesiologist. Outcome data were recorded up to 28 days postoperatively.

**Results:**

The total number of complications was significantly lower in the SG (72 vs. 52 complications, p = 0.038). In particular, infection complications were significantly reduced (SG: 13 vs. CG: 26 complications, p = 0.023). There were no significant differences between the two groups for return of bowel movement (SG: 3 vs. CG: 2 days postoperatively, p = 0.316), duration of post anesthesia care unit stay (SG: 180 vs. CG: 180 minutes, p = 0.516) or length of hospital stay (SG: 11 vs. CG: 10 days, p = 0.929).

**Conclusions:**

This multi-center study demonstrates that hemodynamic goal-directed therapy using pulse pressure variation, cardiac index trending and mean arterial pressure as the key parameters leads to a decrease in postoperative complications in patients undergoing major abdominal surgery.

**Trial registration:**

ClinicalTrial.gov, NCT01401283.

## Introduction

Despite high standards in surgical and anesthetic care in Europe, the perioperative mortality rate is still higher than expected [1]. The aim of goal-directed hemodynamic therapy (GDT), based on the titration of fluids and inotropic drugs to physiological flow-related end points, is to reduce perioperative complications which might even help to reduce perioperative morbidity and mortality [2].

Multiple single-center studies have shown that perioperative GDT may significantly improve outcome, particularly in patients undergoing abdominal surgery [3-5], but also in trauma [6,7] and orthopedic surgery [8]. All these studies were single-center studies which makes the meta-analysis that dealt with these highly divergent studies hard to interpret [9].

The underlying physiological rationale of GDT is that due to improved cardiovascular function, adequate oxygen supply can be maintained intraoperatively. Oxygen debt can be avoided or, if it occurs due to rapid surgical changes such as sudden blood loss, it can be corrected quickly. Routine hemodynamic measurements, such as heart rate and mean arterial pressure (MAP) remain relatively unchanged despite reduced blood flow and are, therefore, considered insensitive indicators of hypovolemia [10] or changes in cardiac index (CI) [11]. GDT is targeted to detect hypovolemia and hypoperfusion early in order to make a quick response possible.

Measurement of blood flow, for example, cardiac output (CO), has traditionally been associated with the use of additional invasive monitoring, including the pulmonary artery catheter or using transpulmonary thermodilution, or less invasively, with the esophageal Doppler. Recently, less invasive devices assessing CO by pulse contour analysis based on the radial artery pressure signal have been introduced [12-15]. Although these devices show lower precision compared to the clinical gold standards of thermodilution, their ability to assess changes in CO adequately is promising [16]. Further, pulse pressure variation (PPV), reflecting the cyclic changes in preload induced by mechanical ventilation, has been shown to reflect accurately volume responsiveness in a number of different high risk surgical groups, thus enabling the avoidance of unnecessary and potentially harmful volume loading [17-22]. GDT based on PPV has also been shown to improve patient outcome [23,24].

We conducted this trial as a multi-center study with the inclusion of a large variety of surgical interventions and patient groups. Standard perioperative care of abdominal surgical patients was compared with hemodynamic management based on PPV and continuous CO trending using radial artery pulse contour analysis. We hypothesized that following this treatment regimen results in reduced postoperative complications (primary endpoint) and reduced length of hospital stay (secondary endpoint).

## Materials and methods

This study was conducted as a multi-center, prospective, randomized, controlled trial between August 2011 and May 2012. Patients were recruited in five centers: Northern State Medical University (Arkhangelsk, Russia), University Hospital Hamburg-Eppendorf (Hamburg, Germany), University Hospital Schleswig-Holstein, (Kiel, Germany), University of Szeged (Szeged, Hungary) and Hospital Clínico Universitario de Valencia (Valencia, Spain). It was approved by the appropriate Institutional Review Boards of all participating centers and conforms to the Journal’s requirements for human trials. It was registered at clinicaltrials.gov with the registration number NCT01401283. Written informed consent was obtained from all subjects.

### Patients

Patients undergoing elective abdominal surgery including general, gynecological and urological surgery were recruited. Inclusion criteria were an anticipated duration of surgery of more than 120 minutes or an estimated blood loss of more than 20% of blood volume, American Society of Anesthesiology (ASA) classification 2 or 3, and an indication for an arterial line and central venous catheter. Exclusion criteria were a planned postoperative high-care intensive care unit stay, pregnant or lactating woman, laparoscopic surgery and arrhythmias. For risk evaluation, ASA classification [25] and Physiological and Operative Severity Score for the Enumeration of Mortality and Morbidity (POSSUM) [26] were documented.

### Enrollment, randomization and blinding

Patients were randomized either to the study group (SG) or the control group (CG), using serially numbered opaque envelopes. Patient enrollment, sequence generation and assignment to interventions were performed by a responsible investigator of each participating study center. Only patients were blinded to group allocation. Care providers and investigators could not be blinded due to the presence of the cardiac index trending monitor.

### Intraoperative management

#### Study group

**Figure 1:**
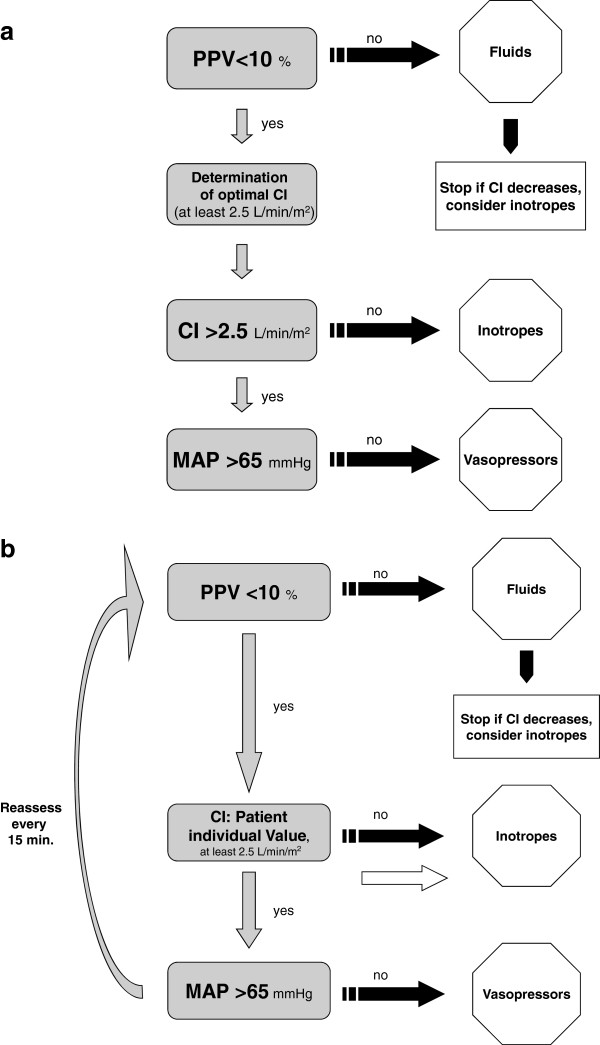
**Hemodynamic treatment algorithms: a) Algorithm for initial assessment and treatment. b) Algorithm for further intraoperative optimization.**

Patients in the SG received basic anesthetic monitoring by five-lead-electrocardiogram, pulse oximetry and blood pressure cuff, at least one peripheral i.v., a central venous catheter and invasive radial arterial blood pressure monitoring. This arterial line was additionally connected to the cardiac index trending monitor (ProAQT, PULSION Medical Systems SE, Munich, Germany). At the beginning of surgery, patients received an initial hemodynamic assessment based on PPV, CI and MAP, as shown in Figure 1a. First, preload was optimized by fluid loading until PPV was <10%. At this point, the patient’s individual preload optimized CI was determined and used as the hemodynamic goal until the end of surgery. Only if this value was below 2.5 L/min/(m^2^), inotropes were applied to reach this minimum CI, serving as a safety parameter to prevent patients from low cardiac output. If PPV and CI were within the target range but MAP was below 65 mmHg, vasopressors were started. After the initial assessment, patients were reassessed every 15 minutes intraoperatively to maintain values according to the study algorithm as illustrated in Figure 1b. Patients were ventilated using tidal volumes of 8 to 10 ml/kg of ideal body weight. Hemodynamic data were documented every 30 minutes, ventilatory parameters every 60 minutes. At the beginning and at the end of surgery blood samples were drawn for arterial and central venous blood gas analysis. At the end of surgery total catecholamine administration, estimated blood loss, urine output and infused fluids were recorded. The time between the end of surgery and extubation was recorded.

#### Control group

Patients of the CG received basic anesthetic monitoring by five-lead-electrocardiogram, pulse oximetry and blood pressure cuff, at least one peripheral i.v., a central venous catheter and invasive radial arterial blood pressure monitoring. Treatment of patients in the CG was entirely performed at the discretion of the care-giving anesthesiologist. Data collection and collection of blood samples for complete blood gas analysis was identical to that of the SG, except for PPV and CI, as patients in the CG did not receive cardiac index trend monitoring.

### Postoperative management

All patients were monitored in the post-anesthetic care unit (PACU) until they were transferred to the ward. Every 15 minutes MAP, central venous pressure and heart rate were recorded. Arterial and venous blood gas analyses were drawn immediately prior to discharge from the PACU, and duration of stay in the PACU was recorded in minutes.

Data on catecholamine use and on estimated blood loss, urine output and infused fluids were obtained 24 hours postoperatively. Return of bowel function, need for enteral feeding, postoperative complications and duration of postoperative hospital stay were recorded for up to 28 days after surgery. The types and clustering of complications were predefined in the study protocol, as described in Additional file 1.

### Endpoints

#### Primary endpoint

Pre-defined postoperative complications for each patient (see Additional file 1) were recorded for up to 28 days after surgery from the patient record and by visiting patients on the ward by the investigators.

#### Secondary endpoint

Length of hospital stay in days was obtained from the patient record.

### Statistical analysis

Sample size calculation was oriented on previously reported data in a comparable single center study of 33 patients [24]. The complication rate in this study was 75% in the CG and 41% in the intervention group. We compared those data with routinely derived data from our study sites, pointing towards an actual complication rate of around 40% in this patient population in our centers. Therefore, the power analysis was on a reduction of complication rate from 40% to 20%, taking into account a power of 80% and statistical significance of *P* <0.05. This revealed a group size of 80 patients per arm. Data were analyzed using Sigma Stat 3.5 and SigmaPlot 10 (Systat Software Inc., San Jose, CA, USA). For continuous data, the Kolmogorov-Smirnov tests were performed to assess normal distribution and, where appropriate, the data were analyzed with the Student’s *t*-test. Non-parametric data were analyzed with the Mann–Whitney *U*-test. Categorical data were compared using *χ*^2^ and Fisher’s exact tests. A level of *P* <0.05 was defined as statistically significant. Data are given in mean ± standard deviation or Median (interquartile range) as appropriate. Comparison of intraoperative data was restricted to the duration of five hours, the time at which 75% of surgeries were finished.

## Results

A total of 180 patients were randomized, ranging from 17 to 43 from each of the five study centers. Twenty patients had to be excluded from the study and/or analysis because of various reasons, listed in Additional file 2. Recruitment for the trial ended after the inclusion of the required 160 patients for analysis. There were 79 patients in the SG and 81 patients in the CG in the final analysis. Demographic data are given in Table 1. Preoperative risk scores, types of surgery and duration of surgery did not differ significantly between the two groups.

**Table 1:** Demographic data

	**Control group (n = 81)**	**Study group (n = 79)**	***P-*** **value**
Age^a^ (years)	65 (18.25)	63 (17)	0.765
Male: female	50: 31	47: 32	0.899
Height^b^ (m)	171.7 ± 9.4	170 ± 9.2	0.241
ABW^b^ (kg)	79.2 ± 18.1	77.4 ± 20.4	0.557
PBW^b^ (kg)	66.0 ± 10.1	64.3 ± 10.1	0.269
ASA III	33	33	0.978
POSSUM physiological^a^	17 (7)	16 (5)	0.921
POSSUM operative^a^	17 (9)	15 (8.75)	0.067
Type of surgery (number of patients)			
Bowel	41	47	0.332
Non-bowel	40	32	0.332
Duration of surgery (minutes)	237.5 ± 109.8	221.9 ± 86.4	0.321

Data are median (IQR)^a^ or mean ± SD^b^. Data were compared using Student’s *t*-test, Mann–Whitney *U*-test, *χ*^2^-test or Fisher’s exact test as appropriate to detect potential preoperative differences between the two groups. ABW, actual body weight; ASA, physical status classification system by the American Society of Anesthesiologists; PBW, predicted body weight: PBW_male_ = 45.5 + 0.91*(height(cm)-152.4), PBW_female_ = 50 + 0.91*(height(cm)-152.4); POSSUM, Physiological and Operative Severity Score for the Enumeration of Mortality and Morbidity.

No specific complications or harm due to the use of the hemodynamics trending monitor or to the application of the study algorithm could be observed.

### Intra- and postoperative hemodynamic parameters

**Figure 2:**
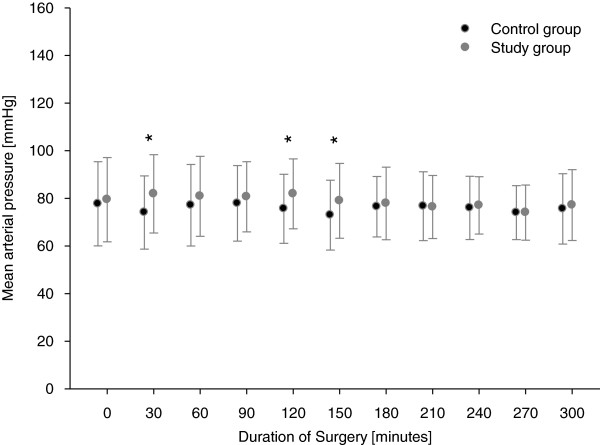
**Mean arterial pressure intraoperatively.** * Significantly different between control group and study group (P < 0.05).

MAP differed significantly at 30 minutes (SG: 81.9 ± 16.4% mmHg versus CG: 74.1 ± 15.4% mmHg, *P* = 0.002), 45 minutes (SG: 83.3 ± 16.2% mmHg versus CG: 74.1 ± 15.1% mmHg, *P* = <0.001), 120 minutes (SG: 81.9 ± 14.7% mmHg versus CG: 75.6 ± 14.5% mmHg, *P* = 0.012) and 150 minutes (SG: 79.0 ± 15.7% mmHg versus CG: 73.0 ± 14.7% mmHg, *P* = 0.025) intraoperatively. These differences are illustrated in Figure 2 and exact values are given in Additional file 3.

Postoperatively, MAP was significantly lower in the CG on arrival to the PACU (SG: 90.5 ± 20.4 mmHg versus CG: 83.5 ± 16.3 mmHg, *P* = 0.024). There were no differences in MAP on discharge from PACU (SG: 90.8 ± 19.0 mmHg versus CG: 88.8 ± 19.9 mmHg, *P* = 0.595).

### Fluids and catecholamines

#### Fluid balance

There were no significant differences in the net amount of fluids administered intra- and postoperatively. Also, urine output and blood loss did not differ between the groups. Values are given in Table 2.

**Table 2:** Fluids intra- and postoperatively

	**Control group (n = 81)**	**Study group (n = 79)**	***P*-value**
**Fluids intraoperatively** (ml)			
Blood loss	704.4 ± 889.6	668.2 ± 676.6	0.773
Urine output	462.0 ± 473.4	414.4 ± 376.4	0.501
Crystalloids + Colloids	3,404.9 ± 1,694.2	3,635.7 ± 1,592.3	0.376
Crystalloids	2,680.2 ± 1,153.8	2,862.0 ± 1,216.0	0.333
Colloids	724.7 ± 720.2	773.7 ± 664.6	0.656
FFP	141.5 ± 620.2	73.7 ± 252.4	0.369
PRBC	224.4 ± 1036.5	144.7 ± 371.6	0.521
Input total	3,770.8 ± 2,827.5	3,854.2 ± 1,954.2	0.829
Fluid balance	2,604.8 ± 2,051.1	2,813.3 ± 1,438.0	0.477
**Fluids postoperatively** (ml)			
Blood loss	249.6 ± 388.8	268.4 ± 324.1	0.780
Urine output	1,679.7 ± 924.2	1,677.3 ± 1,134.6	0.990
Crystalloids + Colloids	3,598.8 ± 2,325.4	3,260.8 ± 2,104.7	0.425
Crystalloids	3,452.2 ± 2,283.2	3,204.2 ± 2,110.9	0.555
Colloids	146.6 ± 307.2	56.6 ± 211.7	0.078
FFP	34.3 ± 189.5	0 ± 0	0.191
PRBC	85.0 ± 379.4	44.8 ± 164.8	0.470
Input total	3,724.2 ± 2,584.2	3,296.0 ± 2,138.2	0.346
Fluid balance	1,724.9 ± 2,374.2	1,357.0 ± 1,871.6	0.373
**Fluids total** (ml)			
Input	7,597.2 ± 4,906.3	7,053.2 ± 3,285.8	0.498
Crystalloids	6,031.5 ± 2,792.6	5,876.9 ± 2,598.2	0.764
Colloids	960.3 ± 862.7	962.6 ± 705.9	0.988
Balance	4,332.6 ± 3,715.7	3,956.5 ± 2,469.7	0.561

Data are mean ± SD. Data were compared using Student’s *t*-test. FFP, fresh frozen plasma; PRBC, packed red blood cells.

#### Vasopressors and inotropes

As listed in Table 3, the number of patients receiving vasopressors was equal between the two groups (SG: 42 patients versus CG: 42 patients, *P* = 0.994), 33 patients in the SG received inotropes during surgery, compared to none in the CG (*P* < 0.001). Few patients required vasopressors postoperatively with no significant difference between the two groups (SG: five patients versus CG: 9 patients, *P* = 0.502). None of the patients received inotropes after the end of surgery.

**Table 3:** Use of inotropes and vasopressors

	**Control group (n = 81)**	**Study group (n = 79)**	***P*-value**
**Inotropes intraoperatively**^**a**^			
Dobutamine	0	33	<0.001
**Vasopressors intraoperatively**^**a**^			
Total	40	37	0.994
Norepinephrine	32	26	0.482
Phenylephrine	4	0	0.135
Ephedrine	8	11	0.584
**Inotropes postoperatively**^**a**^			
Dobutamine	0	0	-
**Vasopressors postoperatively**^**a**^			
Norepinephrine	9	5	0.502

^a^Number of patients.

### Oxygenation

There were no differences in peripheral oxygen saturation between the two groups before surgery (SG: 98.2 ± 1.5% versus CG: 98.1 ± 2.7%, *P* = 0.307), at the end of surgery (SG: 98.2 ± 1.9% versus CG: 98.4 ± 2.1%, *P* = 0.126) and at discharge from recovery (SG: 96.7 ± 2.8% versus CG: 97.1 ± 2.5%, *P* = 0.440). Also, central venous saturation did not show any statistically significant difference before surgery (SG: 82.2 ± 8.4 versus CG: 82.8 ± 7.1%, *P* = 0.649), at the end of surgery (SG: 80.9 ± 7.9 versus CG: 80.9 ± 8.0, *P* = 0.977) and on discharge from the PACU (SG: 73.2 ± 8.9 versus CG: 73.0 ± 7.3, *P* = 0.905).

### Complications

**Figure 3:**
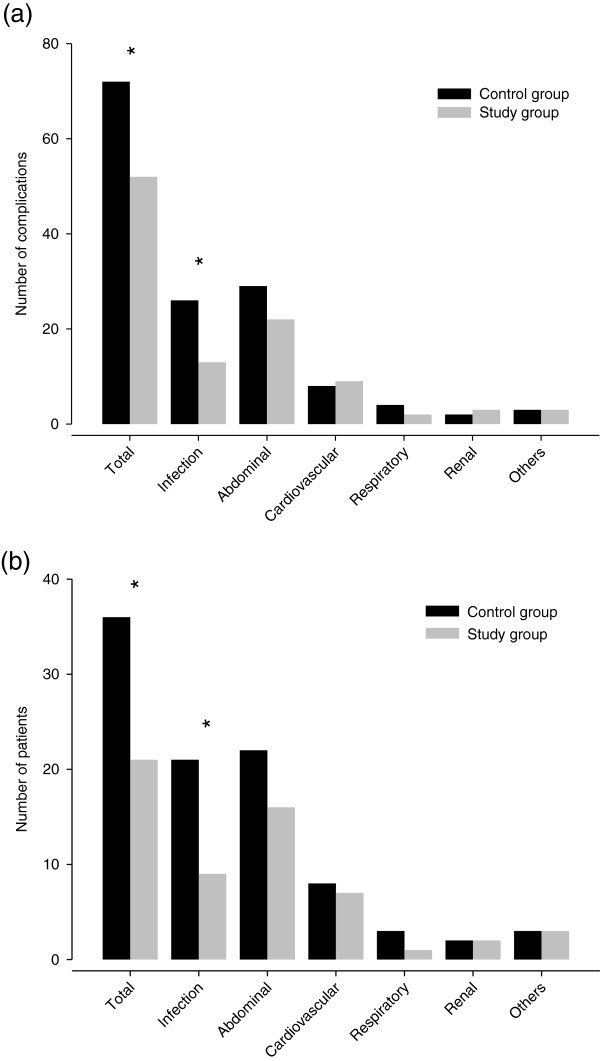
**Postoperative complications. a)** number of complications. **b)** number of patients with complications. * Significantly different between control group and study group (P < 0.05).

The overall number of complications was significantly lower in the SG (52 complications versus 72 complications, *P* = 0.038), as illustrated in Figure 3a. Further, there was a significant difference in the total number of patients with complications. In the SG, 21 (26.6%) patients had at least one complication, compared to 36 (44.4%) in the CG (*P* = 0.028), as depicted in Figure 3b.

Analysis of the predefined clustering of complications showed that there was a significant difference in the subgroup of infection complications (SG: 13 complications versus CG: 26 complications, *P* = 0.023). Also the cluster of abdominal complications showed a trend towards fewer complications in the SG (22 complications versus 29 complications, *P* = 0.328), but without reaching statistical significance, as illustrated in Figure 3a.

**Figure 4:**
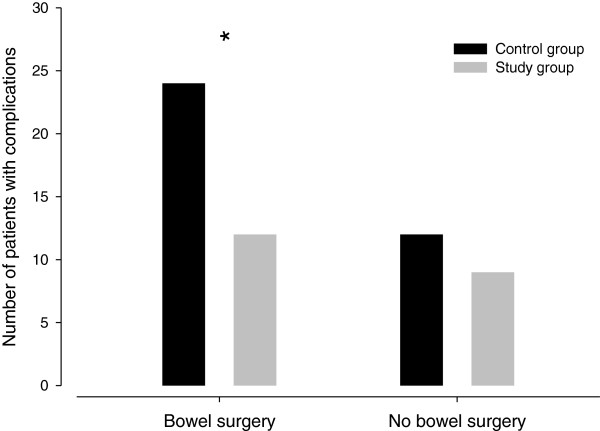
**Number of patients with complications: bowel versus no bowel surgery.** * Significantly different between control group and study group (P < 0.05).

We further analyzed the subgroup of patients who received bowel surgery compared to non-bowel surgery. The reduction in the number of patients with complications was particularly pronounced in the group of patients receiving bowel surgery (SG: 12 complications versus CG: 24 patients, *P* = 0.003) compared to non-bowel surgery (SG: 9 patients versus CG: 12 patients, *P* = 0.931), see Figure 4. Analysis of clustering of complications showed that the number of infection complications in patients undergoing bowel surgery was significantly reduced (SG: 8 complications versus CG: 18 complication, *P* = 0.01).

### Outcome

There were no significant differences in the return of first bowel movement after surgery (SG: 3 (1) days versus CG: 2 (1) days, *P* = 0.316) and the need for enteral feeding postoperatively (SG: 5 patients versus CG: 8 patients, *P* = 0.595). Further, there were no significant differences in the duration of stay in the PACU (SG: 180 (127.5) minutes versus CG: 180 (114) minutes, P = 0.516) or length of hospital stay (SG: 11 (8) days versus CG: 10 (11.8) days, *P* = 0.929) in the two groups.

## Discussion

This is the first randomized multi-center study in patients undergoing major abdominal surgery demonstrating that perioperative hemodynamic GDT using PPV, radial artery pulse contour CI and MAP as its key parameters leads to a reduction in postoperative complications.

Several single-center studies and meta-analyses have already pointed towards the benefit of perioperative hemodynamic GDT on patient morbidity and mortality. These diverse studies with different study protocols and algorithms make comparison and common evaluation very difficult. Furthermore, a single-center study always has the potential limitation that it is performed by a highly skilled study team – this, of course, strengthens the quality of data; however, potentially it does not test the adoption of the protocol into daily clinical practice. Therefore, the intention of our study was to investigate the influence of GDT in different medical centers in different countries, with different local practices and a broad range of different intra-abdominal procedures. In order to keep this diversity and, therefore, allowing its translation in daily clinical routine we did not dictate treatment in the CG.

We chose postoperative complications as the primary outcome parameter because the occurrence of 28-day postoperative complications has been shown to be of greater importance than preoperative patient risk and intraoperative factors in determining survival after major surgery [27]. Undoubtedly, length of hospital stay is one important factor for the individual patient, as well as for the healthcare system. However, it is obvious that it is affected by many aspects besides postoperative complications, including patients’ preoperative fitness and health, but also the social, structural and logistical aspects of each individual patient and each health care system.

The study showed a significant decrease in the primary endpoint (complications) for patients in the SG and also a reduction in the number of patients with complications. This strengthens the results of previous single-center studies [4,5,23,24,28], current meta-analyses [29-31] and a recently published Cochrane review [32]. The review analyzed randomized controlled single center trials on the intervention of increasing perioperative blood flow using fluids with or without inotropes or vasoactive drugs to defined goals in adults. Results are very much in-line with the results of the multi-center study presented here. There was no difference in mortality between the CG and the treatment group. However, the rate of renal failure, respiratory failure and wound infections was significantly reduced in the treatment group. Also, the number of patients with complications was significantly reduced by the intervention [32].

In our study, in particular, infection complications were significantly reduced in the SG. This is in line with a recent meta-analysis demonstrating that perioperative goal directed therapy resulted in a significantly reduced number of surgical site infections, pneumonia and urinary tract infections [33].

A further sub-analysis revealed that, in particular, patients undergoing bowel surgery and treated by GDT seemed to suffer significantly fewer complications. This supports previous results where GDT was found to be of great benefit especially in patients with bowel surgery [4,5]. However, because the study design did not include subgroup-analysis, the power of this study is not sufficient to draw this final conclusion. Further research needs to be directed at high risk patients undergoing major, but not bowel, surgery.

Two very recent trials investigated the effects of GDT in colorectal surgery with different results. Challand *et al*. [34] conducted a double-blinded controlled trial with goal-directed versus standard fluid regimen with subgroups of aerobically fit and unfit patients. They showed that for aerobically fit patients, GDT was associated with no benefit and even had detrimental effects on readiness for discharge and length of hospital stay. We think the reason for these conflicting results is the selection of patients and surgical interventions. The subgroup of aerobically fit patients consisted mainly of ASA 1 and 2 patients. In some cases this results in a combination of a healthy, fit patient receiving low invasive and, in particular, laparoscopic surgery. In these patients we would not expect there to be any great advantages to GDT. That is why for this trial we chose only to investigate major open abdominal surgery and to exclude laparoscopic procedures and ASA 1 patients.

Brandstrup *et al*. [35] conducted a double-blinded, multi-center trial with GDT versus zero fluid balance in open and laparoscopic colorectal surgery showing no differences in length of hospital stay or complications. Again, mostly ASA 1 and 2 patients and also patients undergoing laparoscopic surgery were included.

In our study, although patients from both groups received nearly the same net amount of fluids perioperatively, and also the number of patients being treated with vasopressors intraoperatively was not significantly different between the two groups, 41.8% of patients in the SG received inotropes compared to none in the CG. This is not surprising as the use of inotropes was part of the algorithm for the GDT. As there was no monitoring of CI in the CG, on what could physicians base the decision to use inotropes? In an earlier study, Pearse *et al*. [28], showed a reduction in postoperative complications after the use of GDT postoperatively owing to an increase in global oxygen delivery through volume optimization and inotropic therapy. The authors suggested that through an increase in tissue partial pressure of oxygen there was improved tissue healing and, therefore, a reduction in infection rates. These suggestions are supported by our results. We did not measure oxygen demand or the tissue partial pressure of oxygen but our algorithm was aimed at optimizing intravascular volume and CI resulting in the same effect of improved tissue perfusion and oxygenation. A currently published meta-analysis could not identify evidence for an increased risk of treatment-related cardiac complications following the use of inotropes due to GDT [36], but it is important to note that, if the indication for inotropes is increased on the basis of these findings, special attention will be necessary in high-risk cardiac patients, where current clinical guidelines recommend perioperative ß-blockade as cardioprotection [37]. Although we did not recognize any signs of postoperative myocardial infarction in our patients, further research needs to be directed at this growing subgroup of surgical patients.

Detailed analysis of intraoperative hemodynamic parameters suggests that the timing of fluid loading was better in the SG, as the MAP was significantly higher at four time points of measurement (30, 45, 120 and 150 minutes). This was also the case immediately after arrival at the PACU. These intra- and postoperative data suggest that if there is no algorithm, therapy is undirected and may be delayed because of the anesthesiologists’ fear of fluid overload with its potential harmful consequences.

Measurements of PPV and pulse contour CI were taken via a regular radial arterial line although validation studies of similar devices for monitoring CI have shown varying results. However, although this technique may not provide optimal precision, the CI trending ability seems to be sufficient [12,13,18,38]. The clinical usefulness in hemodynamically stable patients undergoing major surgery is strengthened by the present data. However, if large amounts of blood loss with severe hemodynamic instability are anticipated during the course of surgery, as for example during major vascular surgery or liver transplant, or if the patient is at high risk due to comorbidities, we would recommend additional and more precise monitoring devices for these patients, in terms of a stepwise extension of hemodynamic monitoring.

The intervention in this study, that is, the modification of hemodynamic therapy, was initialized intraoperatively. The rationale was to initiate ‘earliest goal directed therapy’ to maximize the treatment benefit for the whole perioperative course of the patients.

There are several limitations to this study: Only the patients were blinded to their group allocation. Physicians, nurses and investigators could not be blinded because of the use of PPV and CI monitoring and the corresponding algorithms. Furthermore, we decided to omit any additional monitoring in the CG to guarantee that no PPV or CI information could be obtained from the treating anesthesiologist. Certainly, it would have been of interest to have these data for comparison with the SG as well. Since CG care was entirely performed on the discretion of the care-giving anesthesiologist, we assume that CG care was adequate according to clinical expertise and local standards in the participating institutions, which are all high-volume university centers. Of course, we cannot fully eliminate the possibility that the difference in outcomes is not due to benefit in the intervention group but due to poor care in the CG. Sample size calculation was based on the reduction of the total number of complications, which was adopted from earlier studies in this field. We also analyzed the number of patients with complications since this might have been more relevant due to the fact that one patient may develop a large number of complications if they develop multi-organ failure. Further, this study cannot answer the question: is the algorithm proposed here, adding an individualized treatment goal of CI, superior to earlier suggested treatment algorithms? This needs to be answered in future trials. Finally, the results of this study are not transferrable to all patients undergoing major abdominal surgery, since arterial pulse contour analysis and the determination of PPV only works reliably in patients without severe arrhythmias and under fully controlled ventilation.

## Conclusions

In conclusion, this is the first randomized multi-center study on perioperative hemodynamic GDT for patients undergoing major abdominal surgery. The results support a goal-directed therapy approach in order to reduce complications and, therefore, patient morbidity.

## Key messages

● The perioperative use of an algorithm for hemodynamic therapy, based on radial artery pulse contour analysis leads to a reduction of postoperative complications in patients undergoing intra-abdominal surgery.

● The net amount of fluid and volume application is not increased by the application of a goal-directed hemodynamic algorithm based on PPV, CI and MAP.

● The proposed algorithm-driven hemodynamic optimization induced in some patients the additional application of inotropes. It needs to be clarified in future studies, if this application contributed to the reduction in complications.

## Abbreviations

ASA: American Society of Anesthesiology; CG: Control group; CI: Cardiac index; CO: Cardiac output; GDT: Goal-directed hemodynamic therapy; MAP: Mean arterial pressure; PACU: Post anesthesia care unit; POSSUM: Physiological and operative severity score for the enumeration of mortality and morbidity; PPV: Pulse pressure variation; SG: Study group.

## Competing interests

This investigator-initiated and investigator-driven study was supported by an unrestricted research grant from PULSION Medical Systems (Munich, Germany). CS has no other conflicts to declare. JP has no other conflicts to declare. AC has no other conflicts to declare. BB is a board member of PULSION medical systems and received lecture honoraria from Edwars and Deltex. ZM is a board member of PULSION medical systems and Biotest and receives money for consultancy and lectures from PULSION medical systems, Biotest, and Thermofisher. KK has no other conflicts to declare. AH has no other conflicts to declare. JB is a board member of PULSION medical systems. MYK is a board member of PULSION medical systems. SGS is a board member of PULSION medical systems and receives money for consultancy and lectures. DAR is a board member of PULSION medical systems and received lecture honoraria from PULSION and Edwards.

## Authors’ contributions

CS participated in the acquisition of data, analysis and interpretation of the data and drafted the manuscript. JP participated in acquisition of data, analysis and interpretation of the data, helped to draft the manuscript and revised the manuscript critically for important intellectual content. AC participated in acquisition of data and revised the manuscript critically for important intellectual content. BB participated in the design of the study, acquisition of data and revised the manuscript critically for important intellectual content. ZM participated in the design of the study, acquisition of data and revised the manuscript critically for important intellectual content. KK participated in the acquisition of data and revised the manuscript critically for important intellectual content. AH participated in the acquisition of data and revised the manuscript critically for important intellectual content. JB participated in the design of the study, acquisition of data and revised the manuscript critically for important intellectual content. MYK participated in the design of the study, acquisition of data and revised the manuscript critically for important intellectual content. SGS participated in the design of the study and revised the manuscript critically for important intellectual content. DAR participated in the design of the study, acquisition of data, analysis and interpretation of the data, helped to draft the manuscript, revised the manuscript critically for important intellectual content and supervised the research group. All authors read and approved the final manuscript.

## Supplementary Material

Additional file 1 **Types and clustering of complications.** AF, atrial fibrillation; AMI, acute myocardial infarction; CT, computed tomography; ECG, electrocardiogram; MAP, mean arterial pressure; UTI, urinary tract infection; VF, ventricular fibrillation; WBC, white blood cell count. Click here for file

Additional file 2 Patient recruitment. Click here for file

Additional file 3 **Hemodynamic parameters intraoperative.** CI, cardiac index; CVP, central venous pressure; HR, heart rate; MAP, mean arterial pressure; PPV, pulse pressure variation. Click here for file
