# Supplementary material for: Perioperative goal-directed hemodynamic therapy based on radial arterial pulse pressure variation and continuous cardiac index trending reduces postoperative complications after major abdominal surgery: a multi-center, prospective, randomized study
Source: Crit Care. 2013 Sep 8;17(5):R191. doi: 10.1186/cc12885 (PMC4057030; doi:10.1186/cc12885)
Supplement: Additional file 1 — Types and clustering of complications. AF, atrial fibrillation; AMI, acute myocardial infarction; CT, computed tomography; ECG, electrocardiogram; MAP, mean arterial pressure; UTI, urinary tract infection; VF, ventricular fibrillation; WBC, white blood cell count. [file cc12885-S1.doc]

**Additional file 1:** Types and clustering of complications.

| **Infection** |
| --- |
| Respiratory: confirmed chest x-ray, WBC >12x10^3^ or <4x10^3^  ml^-1^ |
| Abdominal – Abdominal CT |
| UTI – dysuria, urine analysis |
| Wound – clinical diagnosis |
| **Respiratory** |
| Ventilation for >24h |
| Unsuccessful extubation |
| **Cardiovascular** |
| Pulmonary edema: auscultation, chest x-ray |
| Arrhythmia: ≥Lown II, ≥30 atrial extra systoles, AF, VF |
| Hypotension: MAP <50mmHg |
| AMI: ECG ischemic signs, troponin T >0.03ng/ml |
| Stroke: confirm with CT |
| **Abdominal** |
| Constipation >4 days |
| Upper gastrointestinal bleed |
| Re-operation |
| **Renal** |
| Urine output <500 ml/day |
| Dialysis for acute renal failure |
| **Other** |
| Massive postoperative bleed |
| Perioperative death |

AF=atrial fibrillation, AMI=acute myocardial infarction, CT=computed tomography, ECG=electrocardiogram, MAP=mean arterial pressure, UTI=urinary tract infection, VF=ventricular fibrillation, WBC=white blood cell count
