# Supplementary figures and images for: Perioperative goal-directed hemodynamic therapy based on radial arterial pulse pressure variation and continuous cardiac index trending reduces postoperative complications after major abdominal surgery: a multi-center, prospective, randomized study
Source: Crit Care. 2013 Sep 8;17(5):R191. doi: 10.1186/cc12885 (PMC4057030; doi:10.1186/cc12885)

**Additional file 2:** Patient recruitment.


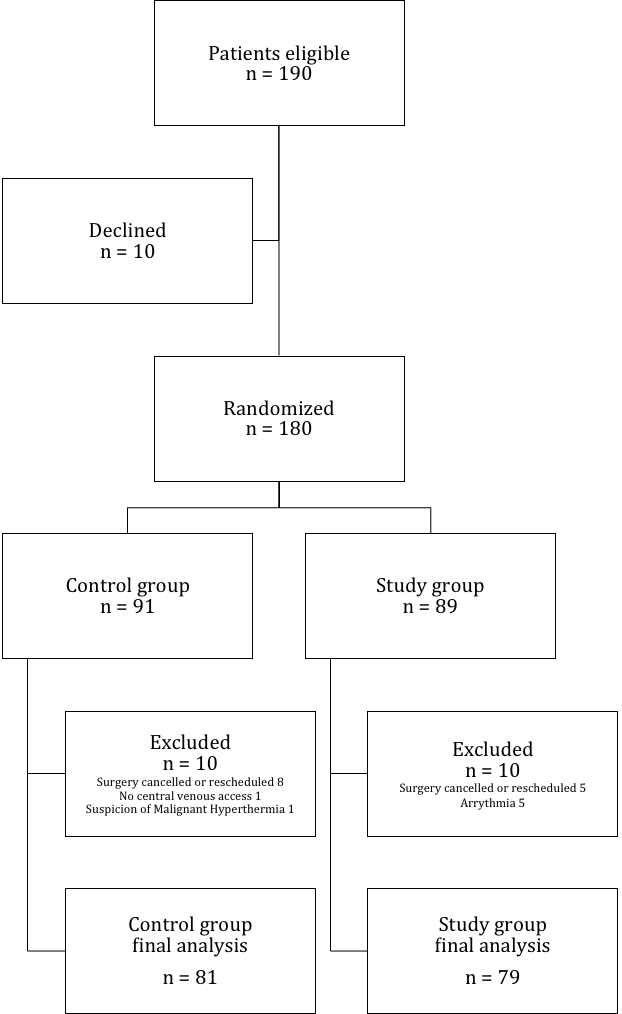

Supplement: Additional file 2 — Patient recruitment. [file cc12885-S2.doc]
