# Supplementary material for: Perioperative goal-directed hemodynamic therapy based on radial arterial pulse pressure variation and continuous cardiac index trending reduces postoperative complications after major abdominal surgery: a multi-center, prospective, randomized study
Source: Crit Care. 2013 Sep 8;17(5):R191. doi: 10.1186/cc12885 (PMC4057030; doi:10.1186/cc12885)
Supplement: Additional file 3 — Hemodynamic parameters intraoperative. CI, cardiac index; CVP, central venous pressure; HR, heart rate; MAP, mean arterial pressure; PPV, pulse pressure variation. [file cc12885-S3.doc]

|  | **Group** | **0 min** | **30 min** | **60 min** | **90 min** | **120 min** | **150 min** | **180 min** | **210 min** | **240 min** | **270 min** | **300 min** |
| --- | --- | --- | --- | --- | --- | --- | --- | --- | --- | --- | --- | --- |
| **HR**  [bpm] | CG | 68.6±16.2 | 67.0±16.4 | 68.3±16.2 | 69.0±14.7 | 67.8±14.1 | 69.7±14.0 | 73.4±12.7 | 75.7±16.6 | 75.5±16.4 | 76.9±17.4 | 79.4±16.1 |
|  | SG | 66.6±14.2 | 68.2±14.1 | 68.4±12.8 | 69.2±13.0 | 68.6±12.5 | 70.7±12.8 | 71.9±12.8 | 71.3±13.4 | 72.9±12.5 | 73.5±13.1 | 76.1±10.9 |
| **MAP**  [mmHg] | CG | 77.7±17.7 | 74.1±15.4 | 77.1±17.1 | 77.9±15.9 | 75.6±14.5 | 73.0±14.7 | 76.5±12.7 | 76.7±14.5 | 76.0±13.3 | 74.0±11.4 | 75.6±14.8 |
|  | SG | 79.4±17.7 | 81.9±16.4 | 80.9±16.8 | 80.6±14.7 | 81.9±14.7 | 79.0±15.7 | 77.9±15.3 | 76.4±13.3 | 77.0±12.0 | 74.0±11.6 | 75.9±12.2 |
| **CVP**  **[**mmHg] | CG | 11.7±5.0 | 11.9±4.8 | 11.3±6.0 | 11.8±5.9 | 11.4±6.1 | 11.2±5.8 | 10.7±5.6 | 10.8±6.3 | 10.0±6.1 | 9.3±6.2 | 8.7±4.2 |
|  | SG | 10.4±3.8 | 11.7±3.7 | 10.9±4.0 | 11.5±5.0 | 11.3±5.1 | 10.6±4.9 | 11.1±4.3 | 11.3±4.1 | 11.5±5.6 | 10.7±4.0 | 11.0±3.9 |
| **CI**  [l·min^-1^·(m^2^)^-1^] | CG | - | - | - | - | - | - | - | - | - | - | ± |
|  | SG | 2.7±0.6 | 3.0±0.8 | 3.0±0.6 | 3.0±0.6 | 3.0±0.6 | 3.0±0.6 | 3.1±0.6 | 3.1±0.6 | 3.4±0.7 | 3.2±0.6 | 3.5±0.6 |
| **PPV**  [mmHg] | CG | - | - | - | - | - | - | - | - | - | - | ± |
|  | SG | 10.4±4.6 | 7.9±4.4 | 8.6±4.0 | 8.5±4.5 | 9.1±4.6 | 9.1±4.2 | 9.4±4.5 | 9.5±5.0 | 8.8±5.0 | 9.4±5.5 | 7.9±4.5 |

**Additional file 3:** Hemodynamic parameters intraoperative.
CI=cardiac index, CVP=central venous pressure, HR=Heart rate, MAP=mean arterial pressure, PPV=pulse pressure variation.
